# Female sexual outcomes in primiparous women after vaginal delivery and cesarean section

**DOI:** 10.4314/ahs.v17i3.4

**Published:** 2017-09

**Authors:** Fatemeh Nasiri Amiri, Shabnam Omidvar, Afsaneh Bakhtiari, Mahmood Hajiahmadi

**Affiliations:** 1 Departments of Midwifery and Reproductive Health, Faculty of Medicine, Babol University of Medical Sciences, Babol, Iran; 2 Departments of Biostatics and Epidemiology, Babol University of Medical Sciences, Babol

**Keywords:** Women's health, cesarean section, post-partum, vaginal delivery, female sexual function

## Abstract

**Background:**

Sexual function is an essential component of life and yet very little is known about the relationships between the female sexuality and the mode of delivery.

**Objective:**

To compare sexual outcomes after vaginal delivery and cesarean section.

**Methods:**

A cross-sectional study was conducted on women in two stages; early pregnancy and 3 to 6 months after delivery in health centers. Female sexual outcomes evaluated were female Sexual Function Index scores and the time required to resume sexual activities after delivery.

**Results:**

Sexual function did not differ significantly among two groups vaginal delivery n=90 and cesarean section n = 113 with regard to duration of marriage, educational level, contraception methods, and occupational status p = 0.8.The mean timing of the resumption of sexual activity was 8.9 ± 1.3, and there was no substantial conflict between the two groups.

Mean frequency of intercourse in the post-partum period was 1.8±1.2 times per week with significant difference compared to pre pregnancy P<0.05. The individual domain scores after the delivery was significantly lower in comparison with pre-pregnancy p<0.004.

**Conclusion:**

No differences in sexual outcomes between vaginal delivery and cesarean section. Consequently cesarean section cannot be recommended in the view of maintenance of normal sexuality after child birth.

## Introduction

Sexual function and its subsequent satisfaction are among the most important aspects of Women's life^1^. Sexual dysfunction in women is defined as the inability to achieve orgasm^2^. Sexual dysfunction refers to difficulties that occur during the sexual response cycle that prevent the individual from experiencing satisfaction from sexual activity. It is relatively difficult to estimate the prevalence of female sexual dysfunction (FSD), because the definition and diagnostic criteria are still controversial and under development.^3^ The American Foundation for Urologic Disease has confirmed four types of female sexual dysfunction: low libido, problems with sexual arousal, inability to achieve orgasm, and dyspareunia^4^.

Reasonably valid results for studies on prevalence of women's sexual dysfunctions were obtained from 18 descriptive epidemiological data indicating that about 40–45% of adult Women and 20–30% of adult men have at least one manifest sexual dysfunction rather often, often, nearly always, and always^5^. The prevalence of female sexual dysfunction in Iran is approximately 31%^6^. Despite the fact that there is a high prevalence in female sexual dysfunction, there have been few research studies investigating female sexual problems^7^.

The accurate prevalence of female sexual dysfunction is unknown in our community, but the prevalence of sexual dysfunction in American men and women were 31% and 43% respectively; according to the report released by National Health and Social Life Survey^8^.

Lack of libido, inability to reach orgasm and sexual pleasure, and pain during sexual intercourse are among the most common sexual problems women usually suffer from^9^. It has also been shown that the frequency of sexual activities during pregnancy^10^ and post-partum^11^ could be reduced, but the results are by no means consistent. Several studies have also shown that sexual problems are more common among women with pelvic floor muscle disorders^12^–^14^. It is worth mentioning that a lot of biological and psychosocial changes can occur during pregnancy and the post-partum period, but unfortunately not all women adapt well to these changes^15^–^17^.

There is a positive relation between the severity of dyspareunia and perineal trauma in particular, episiotomy^18^–^20^. Some women prefer caesarean section CS to vaginal delivery as they believe that they will have a smaller chance of dyspareunia in their postpartum period; but obviously there is still doubt that the mode of delivery is associated with female sexual function^18^,^19^,^21^.

Normally, women undergo caesarean section in case a known medical condition is threatening, the life of the mother or the baby^22^. But nowadays the tendency of women, with no medical indications, to CS is on the rise, making CS a popular mode of delivery around the globe^23^. It is still a controversial issue whether CS should be performed under maternal request or the physicians' recommendation^24^. The World Health Organization (WHO) has announced the rate of CS is on average 10–15% in the world^25^. The rate of CS in Iran has experienced a sixfold increase in the last three decades^26^. The Demographic and Health Survey (DHS) conducted in Iran in 2000 reported the C-section rate in the country to be as high as 35%^27^. These findings are congruous with the previous results, shedding light on the fact that the rate of CS in Iran is increasingly on the rise^27^,^28^. The ‘Integrated Monitoring Evaluation System’ Survey (IMES) conducted on urban and rural populations of Women, as representative of the Iranian population, also reported the rate of cesarean-section to be as high as 40%, which is higher than the rate reported in developed countries such as the US (30.2%) and England (22%). The rate is believed to be comparable with that of fast economically growing countries such as Brazil (41.3%) and China (40.5%)^29^.

There has also been a long-standing controversy over what is the best mode of delivery VD Vs CS to diminish the risk of post-natal morbidity, which has not only affected the professionals' perspectives to the issue, but also changed the way women looked at the childbirth experience^30^.

Despite all the controversies and the significance of the issue, insufficient research has been conducted so far to pinpoint the problem more accurately. The present study aimed to compare the post-partum sexual function in primi-parous women after vaginal delivery and caesarean section in Babol.

## Materials and methods

This cross-sectional prospective study was conducted on women with antenatal normal pregnancy at the first trimester and was followed until post-delivery. This study was conducted in health centers affiliated to Babol University of Medical Sciences in Babol, Iran, from June 2011 to September 2012. The area of health care centers related to Babol University of Medical Sciences in Babol was divided into four zones, namely North, South, West and East, and then one center was selected from each zone randomly. The health center was considered as a cluster. The clusters were selected randomly and quota based on the number of pregnant women receiving prenatal care was considered.

Eligible women presented to the above health centers were randomly selected for participation in study.

The number of samples needed for the mean female sexual function scores in women with vaginal delivery and caesarean section (180) n = 90 per group were estimated by α= 0.05, Zα=1.96, Z beta=1.28, Beta=0.10. But as this study was prospective and risk of sample loss exists, we enrolled 240 nulliparous pregnant women who were referred to health centers and were eligible to participate in the study were selected. Participants were selected by a two stage sampling. A total of 240 pregnant women who were referred to the health clinics at Babol University of Medical Sciences were recruited consecutively. Eligible women also presented to the above health centers were selected for participation in the study, so 228 Women met the study criteria, and of whom, 203,113 with CS and 90 with NVD completed the whole study period. In the first stage of study 228 pregnant women who referred to receipt prenatal care were questioned about their pre-pregnancy Sexual Function. In the second stage of study the women who had brought their babies to same health clinics for immunization 3 to 6 months after delivery (n=203) were questioned about their post-partum sexual function. 25 of the women (10.1%) did not participate in the second stage sampling for various reasons for example some women refused or had moved to other cities.

The inclusion criteria for the study were: receiving prenatal care in the first trimester and then bringing their babies to the same health clinics for immunization within 3 to 6 months of delivery, negative history of instrumental vaginal delivery. In our area hospitals, all of Nulliparous women with vaginal delivery had an episiotomy commonly.

The data collection tool was a questionnaire, completed by the researchers through interviews. If subjects had symptoms of vaginitis, they were excluded because of the effect of Vaginitis on the sexual function. So the participants with the following criteria were excluded From the study: abortion and stillbirth or perinatal child loss, birth neonate with anomalies, preterm delivery, previous pelvic surgery, the history of previous marriage, the history of sub-fertility, the body mass index of > 30, consuming medications with adverse effects on sexual function e.g, blood pressure lowering drugs, anti-arrhythmic drugs, sedatives and tricyclic antidepressants, having physical and mental problems, the presence of relationship problem with their spouse, mental retardation, smoking and alcohol consumption, and having a critical misfortune such as the death of relatives in the past year.

Informed consent was obtained from each participant through the provision of an information leaflet coupled with verbal reassurance that participation was entirely voluntary and that the participant could withdraw at any time. All participants were assured in terms of confidentiality and anonymity. In addition, all participants were requested to put their responded questionnaires in a covered box.

Data collection tool was a questionnaire completed through interviews in private meetings by the researcher. The questionnaire comprised of two parts: Socio-demographic characteristics age, level of education, employment, and the partners' age, job, and level of education and obstetric history, type of birth, episiotomy, parity, infant's gender, birth weight of infants, contraceptive methods used and gynecological status, the symptoms of vaginitis, breastfeeding and other information the time for the resumption of sexual intercourse and the frequency of sexual contacts per week. The second part of the questionnaire was related to sexual functioning prior to pregnancy and the period of 3 to 6 months postpartum. The Sexual function was measured on the basis of a valid and reliable Female Sexual Function Index (FSFI) questionnaire. FSFI contains 19 items and addresses women's sexual function in 6 dimensions including sexual desire frequency and level of sexual desire, arousal frequency and level of arousal and satisfaction with sexual arousal, lubrication frequency and difficulty of vaginal lubrication and problems in orgasm related to vaginal lubrication, orgasm frequency of orgasm, difficulty in reaching orgasm and satisfaction with orgasm, satisfaction with emotional closeness with a spouse, satisfactory sex with the spouse and the overall life satisfaction and pain frequency and level of pain during and after intercourse as a generic standard questionnaire. A higher score in each domain indicates better status^7^. The minimum and maximum score of each domain included: desire (2–10), arousal (0–20), lubrication (0–20) orgasm (0–15), satisfaction (2–15), pain 0–15. The Psychometric properties of the questionnaire were confirmed by other studies^7^, ^31^. The Validity and reliability of the Iranian version of FSFI questionnaire on women with sexual dysfunction and control groups has been reported in a study by Mohhamadi et al in Iran. The Cronbach's alpha for the sexual function index and its dimensions ranged between 0.70 sexual desire to 0.91 orgasm^32^. The construct validity of the questionnaire established And reported in a study by Khademi et al on 547 Iranian women indicated 5 dimensions^33^, which were comparable with the original factors in a study by Rosen et al^7^. The data was analyzed by descriptive and inferential statistics. The analysis of findings was done by the Statistical package for social sciences SPSS V.18 software. P values of less than 0.05 were considered significant.

Differences in variables between groups were determined with the Students two tailed t test And for dichotomous and normally distributed continuous variables, respectively. Proportions were compared using Chi-Square test. Analysis of variance was used to test for significance of difference between means in groups.

## Ethical considerations

This study was approved by the Ethics Medical Research Committee of Babol University of Medical Sciences. The participants had given written informed consent before the study commenced. The use of numbers ensured the confidentiality of the task, and no names appeared on the questionnaires.

## Results

The present study was conducted on a total number of 203 primiparous women, 90 women who had episiotomy had NVD, and 113 women who had CS within 3 to 6 months of their delivery. The socio-demographic characteristics of the women are shown in Table 1. The age range of the participants was 18 to 40 years. The mean age of the women was 24.91 ± 4.9, and the mean age of their husbands was 29.9 ± 5. 7. The length of their marriage was 5.93 ± 5.4 years on average. All of them were breast-feeding their infants. According to the data, the scores related to sexual function did not differ significantly among two groups NVD (n = 90) and CS (n=113) with regard to duration of marriage, educational level, contraception methods, and occupational status (P>0.05). The birth weight of the neonates Was between 2500 to 4000 grams in 82.9% of the cases. There was also no difference between the groups with regard to the birth weight of their neonates Table 1.

**Table 1:** The socio-demographic characteristics of the Women 3 to 6 months after delivery with NVD and CS (N=203)

Characteristics	Women with NVD (n=90) Number (%)	Women with CS (n=113) Number (%)	P value
Educational level: High school or higher Primary School	66(75.0) 24(25.0)	84(73.4) 29(26.6)	*N.S
Occupational Status: Employees Housewife	66(75.9) 21(24.1)	86(76.1) 27(23.9)	N.S
Sex baby: Boy Girl	46(51.1) 44(49.9)	60(53.1) 53(46.9)	N.S
Birth Weight: <2500 2500–4000 >4000	5(5.6) 75(83.3) 10(11.1)	7(6.2) 93(82.3) 13(11.5)	NS
Contraception Methods after delivery: Condom Withdrawal mini-pill intrauterine device other contraceptive	38(43.2) 32(36.4) 15(17) 2(2.3) 1(1.1)	48(42.5) 40(35.5) 18(15.9) 4(3.5) 3(2.6)	NS

The comparison between pre-pregnancy before the pregnancy started and post-partum 3 to 6 months after delivery female sexual function scores are shown in Table 2. The mean ±SD score of female sexual function with regard to desire, arousal, orgasm and satisfaction within the period of 3 to 6 months of delivery was significantly lower than that of prepregnancy, p<0.004. Significant decreases in the test scores were also observed for all the key dimensions of sexual function desire, arousal, orgasm, and satisfaction.

**Table 2:** The FSFI scores in pre pregnancy and 3 to 6 months after delivery

Domain	pre pregnancy	postpartum	P value
**Desire**	7.0 ± 1. 7	5.9 ± 1. 7	**0.001**
**Arousal**	6.6 ± 1. 7	5.9 ± 1. 7	**0.001**
**Orgasm**	7.5± 1. 6	7.0 ± 1. 7	**0.001**
Pain	6.9 ± 2.0	6.8 ± 2.0	0.4
Lubrication	7.5± 1. 5	7.5± 1. 6	0.48
**Satisfaction**	8.2± 1. 4	7.9± 1. 6	0.004

The maternal age and the sexual orgasm before pregnancy and after delivery were found to be inversely correlated R= − 0.19, P=0.02. But there was no significant difference between the ages of their husbands. The mean ± SD for self-reported timing of the resumption of sexual activity was 8.9 ± 1.3. There was no significant difference between the two groups p= 0.66. Also, there was no significant relation between the onset of the resumption of sexual activity and the birth weight of the neonates. The FSFI scores of the two groups and the comparison between them are given in Table 3.

**Table 3:** The FSFI scores in NVD and CS groups within 3 to 6 months after delivery

Domain	NVD(N=90)	CS(N=113)	P value
**Desire**	5.9 ± 1. 7	6.1 ± 1.5	P=0.20
**Arousal**	5.9 ± 1. 6	5.8 ± 1. 7	P=0.35
**Orgasm**	7.1 ± 1. 4	6.9 ± 1. 8	P=0.19
Pain	6.8 ± 1.5	6.7 ± 1. 7	P=0.34
Lubrication	7.0 ± 1. 6	7.1 ± 1. 5	P=0.33
**Satisfaction**	6.8 ± 1. 2	6.9 ± 1. 2	P=0.50

There was also no statistically significant difference between the sexual function scores of the two groups, NVD and CS (p = 0.8). In overall the methods of contraception in 3 to 6 Months post-partum were: condoms (42.2%), withdrawal (35.7%), mini-pill (16.1%), the intrauterine device, IUD, 1%, and other contraceptive methods (3%). The percentage of Women who went without contraception was 2 %. It should be noted that no relation was found between the method of contraception in post-partum and the sexual function scores.

The increase in dyspareunia was related with the decrease in orgasm in the pre-pregnancy, R= − 0.34, p =0.002 as well as the post-partum periods R= − 0.13, p = 0.037. The mean coitus in post-partum period was 1.84±1.20 per week. There was a significant difference between the pre-pregnancy and the post-partum period in the two groups p<0.05.

## Discussion

The results of the current study showed that there was no difference between primiparous women who had vaginal delivery through mediolateral episiotomy and those who experienced cesarean section with regard to their sexual function. This finding is in line with a study of Baytur et al, which concluded that the mode of delivery had no impact on the sexual function of women^34^, but this result seems to be incongruous with that of Lydon-Rochelle et al^35^ which concluded that within 7 weeks of post-partum, women who had undergone CS, or had experienced instrument-assisted vaginal labors had significantly lower general health and sexual function scores compared with those who had normal vaginal delivery. Also Qian R et al 36 concluded that caesarean delivery resulted in a greater incidence of adverse effects on post-partum sexual function compared to vaginal delivery.

Signorello et al^37^ proposed that the use of obstetric instruments and the degree of perineal trauma positively relates with the severity of post-partum dyspareunia, impaired sexual sensation, sexual satisfaction, and the ability to achieve orgasm. But these findings largely depend on the short-term changes in post-partum sexual function.

Furthermore, the decrease in post-partum sexual function in women may also be attributed to a hypo-estrogenic state that can occur due to lactation; emotional and relational changes such as the changing body image; fatigue brought about by the baby's needs; and the quality of the relationship with her spouse^34^. The present study, however, proved that there was no difference between the two groups in terms of long-term sexual dysfunction, and that the sexual function of women does not seem to be related to the mode of delivery.

Another study by Lagana et al, demonstrated that female sexual dysfunction in patients who underwent episiotomy during delivery markedly led to low FSFI scores^38^. There is a little consensus among researchers as to whether episiotomy can specifically predispose women to sexual dysfunction. In a longitudinal prospective study, the delivery mode and the episiotomy were reportedly not associated with anorgasmia in primiparous women^39^.

Also, according to the systematic review evaluating the outcomes of routine episiotomy, no evidence was found to support the fact that episiotomy reduced impaired sexual function^40^. In a randomized study of vaginal delivery versus elective CS for singleton fetus in breech presentation, Hannah et al, suggested that there was no difference in sexual outcome within 3 months to 2 years post-partum outcomes^41^.

The sexual function is significantly decreased within 3 to 6 months of the childbirth compared with the pre-pregnancy period. These differences between pre-pregnancy and post-partum scores seemed to be mostly due to desire, arousal, orgasm, and the satisfaction domain of the test.

This finding is consistent with those of Asselmann, E et al^42^, Walraven et al^43^, and Barrett et al^44^ which suggested that the sexual function in women, within 3 to 6 months of delivery, was significantly lower than that of pre-pregnancy period. Breast-feeding seems to be closely associated with the poor postpartum sexual functioning in women^45^. Since the participants of the present study were still breast-feeding their babies, such concerns are not applicable to the population of this study. According to previous studies, the average onset of sexual intercourse, is 5 to 8 weeks after the delivery^46,47^, but the results of this study showed that the mean time for the resumption of sexual intercourse was 8.95± 1.3 weeks, Which is in line with the result of a study by Anzaku and Mikah^48^. What makes the present study distinct from the previous studies is the use of a validated sexual function instrument in a prospective, multicenter study with carefully characterized obstetric patients. Like any other studies, this study is not without limitations. Sexual function is very complicated and is affected by many factors, including the person's lifestyle, interpersonal relationships and cultural conditions.

Some pregnant women preferred CS, fearing that vaginal delivery affects sexual dysfunction. In Iran more and more pregnant women want an elective caesarean delivery because of fear of dyspareunia after vaginal delivery. Fortunately in this study, most of the women reported “enjoyment at sexual intercourse” within a time period of 3 of 6 months after delivery. This answer was not dependent on the mode of delivery. We believe post-partum sexual counseling should be a part of antenatal follow-up. In conclusion that no differences in sexual outcomes between vaginal delivery and cesarean section. The caesarean section shouldn't be advised for maintaining normal sexual function after delivery.
